# Complete Genome Sequence of *Luteibacter pinisoli* MAH-14

**DOI:** 10.1128/MRA.00774-19

**Published:** 2019-07-18

**Authors:** David A. Baltrus, Meara Clark, Patrik Inderbitzin, Daniela Pignatta, Victoria Knight-Connoni, A. Elizabeth Arnold

**Affiliations:** a School of Plant Sciences, University of Arizona, Tucson, Arizona, USA; b School of Animal and Comparative Biomedical Sciences, University of Arizona, Tucson, Arizona, USA; c Indigo Agriculture, Charlestown, Massachusetts, USA; d Department of Ecology and Evolutionary Biology, University of Arizona, Tucson, Arizona, USA; University of Maryland School of Medicine

## Abstract

Diverse strains of Luteibacter (Gammaproteobacteria) have been isolated from a variety of environments, most frequently in association with both plants and fungi. Motivated by the lack of genomic information for strains throughout the genus *Luteibacter,* we report here a complete genome sequence for Luteibacter pinisoli strain MAH-14.

## ANNOUNCEMENT

Strains classified within the genus *Luteibacter* (*Gammaproteobacteria*) are yellow-pigmented members of microbiomes found in close association with plants (1–4). A subset of strains have also been characterized as endohyphal bacteria within a diverse set of ascomycete fungi (5–8). These bacterial/fungal complexes modulate diverse fungal phenotypes, including the potential for some fungi to act as plant growth promoters (5–8). To increase the amount of genomic information for strains in this genus, we have sequenced a species of *Luteibacter* originally isolated from rhizosphere soil of Pinus koraiensis (2). Here, we report the complete genome sequence of *Luteibacter pinisoli* strain MAH-14.

An isolate of strain MAH-14 (KACC 19298) was acquired from the Korean Agricultural Culture Collection (KACC) and streaked to single colonies onto lysogeny broth (LB) agar plates. Plates were incubated at 27°C for 3 days until single colonies were apparent. A single colony was transferred to LB liquid medium and frozen in glycerol as part of the Baltrus lab culture collection (DBL1436). A sample of this frozen stock was independently streaked onto LB agar plates for each DNA extraction. Single colonies were then picked and transferred to 2 ml of LB broth and grown overnight at 27°C in a shaking incubator at 220 rpm. Genomic DNA used for Illumina sequencing was isolated from a 2-ml overnight culture via the Wizard kit (Promega, Madison, WI) following the manufacturer’s protocols. Genomic DNA used for Nanopore sequencing was isolated from a separate 2-ml overnight culture via a Nanobind kit (Circulomics, Baltimore, MD) following the manufacturer’s protocols. RNase was added per the manufacturer’s protocols for each of the genomic isolations.

Genomic DNA was sequenced by SNPsaurus (Eugene, OR) using an Illumina HiSeq 4000 instrument and following their standard workflow for library preparation and read trimming. This workflow uses a Nextera tagmentation kit for library generation, followed by sequencing that generated 150-bp paired-end reads, followed by trimming of adaptors from reads with the computational suite BBDuk (BBMap version 38.41) (9). This workflow generated a total of 2,707,288 trimmed paired reads and 817.6 Mbp of sequence (∼177× coverage).

A separate isolation of genomic DNA was sequenced by the Baltrus lab via an Oxford Nanopore MinION device with 1 μg of DNA prepared using the LSK-109 kit without shearing. Reads were called during sequencing using Guppy version 2.0.10, and only the reads which passed the initial quality check (QC) during the run (and which were therefore placed into the “fast5_pass” folder) were used. Sequencing on the MinION device generated 4,000 reads for a total of 42.8 Mbp of sequence (∼10× coverage).

Hybrid assembly of all read types was performed using Unicycler version 0.4.8 (10) and resulted in a single chromosome of 4,610,686 bp of sequence with a 66.1% GC content. Unicycler also specified that this chromosomal assembly was circular. This chromosomal sequence was annotated by the NCBI Prokaryotic Genome Annotation Pipeline (PGAP) version 4.8 (11), and it is predicted to contain 4,175 genes representing 4,081 predicted protein coding sequences, 2 complete sets of rRNAs (5S, 16S, and 23S rRNAs), 51 tRNAs, and 4 noncoding RNAs (ncRNAs). Default parameters were used for all software.

### Data availability.

This genome project is indexed at GenBank under BioProject accession number PRJNA548583. The complete genome sequence for *Luteibacter pinisoli* can be found under GenBank accession number CP041046, and the reported genome is the first version, CP041046.1. Trimmed Illumina reads can be found under SRA run accession number SRR9305630. Fast5 files from the MinION run, which passed the initial QC during the MinION run and were therefore placed into the “fast5_pass” folder, can be found under SRA run accession number SRR9305631. A log file generated by Unicycler for an assembly of this genome can be found at figshare (https://doi.org/10.6084/m9.figshare.8330105.v1).
